# The Benefits of vitamin E on liver function and the hemopoietic System in thalassemia Patients

**Published:** 2012-09-22

**Authors:** Z Hashemian, A Hashemi, M Fateminasab

**Affiliations:** 1 Department of Pediatrics, Hematology, Oncology and Genetics Research Center, Shahid Sadoughi University of Medical Sciences and Health Services, Yazd, Iran.; 2 Shahid Sadoughi University of Medical Sciences and Health Services, Yazd, Iran.

**Keywords:** Vitamin E, Hemopoietic System, Liver Function Tests

## Abstract

**Background:**

β−Thalassemic children have oxidative stress and antioxidant deficiency even without iron overload status. In these patients, tissue damage due to oxidative stress may be occurred. Also, it seems that thalassemic patients have higher levels of ALT, AST therefore, the main aim of the present study was to determine the benefits of vitamin E as an antioxidant supplements in β-Thalassemia children.

**Materials and Methods:**

This clinical trial was carried out on 45 beta-thalassemic patients undergoing occasional transfusions (24 males, 21 females), mean age 16± 8 years, admitted to Yazd and Shahid Sadoughi hospital in 2011. Fallowing three months treatment of vitaminE (vitamin E 400-600 unit/day),liver function test and hemopoitic system parameters were measured.

**Results:**

Fourty five patients with laboratory confirmation of β-Thalassemia were recruited following three months vitamin E supplementation, liver function test had higher improvement compared to hemopoitic system parameters , and also serum SGOT was significantly reduced (P-value<0.004 ).

**Conclusion:**

It seems clear that treatments of β-thalassemic patients with vitamins E have benefits in promoting antioxidant status and may improve liver function test, as AST and ALT to decrease but this supplement is not effective for hemopoietic system variables.

## Introduction

In β-thalassemia syndromes, decreased or impaired biosynthesis of beta globin leads to accumulation of unpaired alpha globin chains. Excess presence of the alpha globin chains is the primary reason for the cellular oxidative damage in thalassemia (1).

The prevalence of thalassemia gene in the world is about 3%. About 15,000 people are known as thalassemic in Iran, and about 3,000,000 people are carrying thalassemia gene (2).

The patients with β-thalassemia major usually suffer from iron overload as a consequence of recurrent transfusion and ineffective erythropoiesis. Iron has a catalytic role to produce powerful reactive oxidant species (ROS) and free radicals, which lead to oxidative damage (3,4,5,6). Β-Thalassemia children have oxidative stress and antioxidant deficiency even without iron overload status (7).

 Patients with β-thalassemia are mainly exposed to oxidative stress due to iron overload. Therefore evaluation and maintenance of antioxidant defense can be useful in protecting β-thalassemia patients from more serious complications of the disease (4,8,6,9). In beta thalassemia major, tissue damage occurs due to oxidative stress, and it happens because of the accumulation of iron in the body. Spontaneous oxidation of unpaired alpha-globin chains leads to production of super oxide ions and hydrogen peroxide (10, 8, 11, 12). 

Red blood cells in thalassemics have morphologic abnormalities, which result in increased susceptibility of thalassemic red cells to the exogenous peroxidant threat. Two mechanisms for inadequate peroxidant defense in thalassemics are insufficient vitamin E levels in red blood cells and plasma, and decreased activity of several enzymes including SOD and GPX, which are the first line of defense against oxidant stress. In a study in Italy, a significant decrease of Total Antioxidant Capacity (TAC) in thalassemic patients was reported compared to the control groups (13). 

The antioxidant activity of vitamin E has persuaded many groups to study its ability to prevent chronic diseases, especially those believed to have an oxidative stress component such as cardiovascular diseases, atherosclerosis, and cancer (14,15). 

## Materials and Methods

We have studied 45 patients aged 3 to 45 years with thalassemia (mean age 16± 8 years), who were consecutively referred to the Shahid Sadoughi hospital in Yazd , from June 2011 to June 2012 .The study was approved by the Shahid Sadoughi Hospital ethic committee. The diagnosis of β-thalassemia major was made considering the results of hemoglobin electrophoresis and clinical features of the patients. Twenty three boys and 22 girls were diagnosed in which 17 of them were more than18.

Treatment duration was 3 months, and 12 blood transfusions were regularly conducted for all patients with 10-15 ml/kg packed cells, every 2 to 4 weeks for maintaining hemoglobin levels above 9.5 g/dl; All the patients were under iron chelation therapy with subcutaneous deferoxamine (DFO) 20-60 mg/kg/d. So the patients were under regular blood transfusion and regular chelation therapy during of study. Patients were under chelation therapy with deferoxamine (DFO) at least five times a week, as an overnight subcutaneous infusion (8 to 12 hours). The vitamin E therapy in thalassemic patients started immediately after the first transfusion (after selection of the patients). Each parameter (Hb, Hct, PT, PTT and liver function test) were determined before and after three months treatment (vitamin E 400-600 unit/day) in all of the patients. An oral daily Supplementation 400 milligram of vitamin E in patient weighed less than 20 kilograms and 600 milligram of vitamin E in patient weighed at least 20 kilograms were given for 3 months (7). Five milliliter of blood was taken from each patient following 8-12 h fasting. Sampling was performed just before the transfusion. Blood was drawn from the patients each time just before they received transfusion when hemoglobin values were at their lowest. The exclusion criterias were acute or chronic infection, other hematologic disease, malnutrition, antioxidant or herbal medicine taking, and patients who were suspected to acquire vitamin E allergy.

**Statistical Analysis**

Data were analyzed using descriptive and inferential statistics. The differences in the continuous variables were compared by using the paired-T test (SPSS for Window version 11.0, Chicago, USA). A P-value of <0.05was considered statistical significant. Data are presented as means ± SD.

## Results

In this clinical trial study, Fourty five (24 males, 21 females) thalassemic patients with mean age 16± 8 years who had inclusion criterias to enter the study, were randomly selected. Several variables were measured prior and after 3 months in subjects. The levels of hemoglobin (Hb), hematocrit (Hct), red blood cell (RBC) count, SGOT, SGPT, BS, PT, PTT are shown in Table I. Although thalassemic patients did not show any improvement in Hb, HCT and RBC count, after vitamin E treatment, serum SGOT was significantly reduced (P-valu<0.004 ). The basal RBC count, Hct, and Hb decreased after supplementation, the data suggested that vitamin E didn’t have any effect on these variables .The liver function test had higher improvement compared to hemopoietic system changes. There was no significant correlation between variable in two groups before and after treatment. The changes in the levels of Hb, Hct, RBC count, SGOT, SGPT, BS, PT and PTT in the blood of the two groups are summarized in Table II. In each subgroup, multiple comparisons between means of serum ALT, AST, Hb, Hct, RBC count were assessed. As table II shows, there was no any significant change in the groups following supplementation. There was no any correlation between the age of the patients and the hematological parameters; as well as any other parameter examined throughout this study. In the present study, we found that hematological and characteristics of our thalassemic patients was not improved except for serum SGOT.

**Table I:** Mean (SD) of variables before and after treatment

P-value	after Mean ± SD	before Mean ± SD	variable
Hb	8.83 ± 1.31	8.37 ± 1.10	0.023
Hct	27.19 ± 3.94	26.38 ± 3.36	0.194
RBC	3.50 ± 0.52	3.29 ± 0.48	0.018
SGPT	39.29 ± 40.04	30.25 ± 24.85	0.154
SGOT	41.05 ± 34.71	28 ± 16.04	0.004
BS	94.09 ± 16.26	88.16 ± 18.15	0.056
PT	12.5 ± 1	13.02 ± 0.99	0.009
PTT	38.18 ± 9.28	36.15 ± 7.07	0.118

**Table II:** Comparison variation of hematologic in two age group

** age**	**<=18**		**>18**		**P-value**
**variable**	**Mean**	**SD**	**Mean**	** SD**	
**Hb**	- 0.47	1.54	- 0.43	0.79	0.936
**Hct**	- 0.55	4.72	- 1.23	2.86	0.593
**RBC**	- 0.21	0.64	- 0.20	0.40	0.941
**SGPT**	- 17.74	42.05	4.76	37.22	0.078
**SGOT**	- 16.37	33.97	- 7.44	11.15	0.217
**BS**	- 6.21	20.63	- 5.43	19.55	0.903
**PT**	0.43	1.15	0.66	1.43	0.561
**PTT**	- 3.16	7.76	- 0.23	9.41	0.268

## Discussion

Several studies have been performed to assess antioxidant defense in thalassemic patients. An increased oxidant stress and a decreased antioxidant status promote per oxidative damage to cell and organelle membranes. It is well documented that disturbances of oxidant-antioxidant balance occur in hemoglobinopathies, especially in thalassemia and sickle cell diseases. In majority of these studies, a decreased serum vitamin E was reported (4,8,16,17,18). 

In a study by Rachmilewitz E.A et al showed that the mean serum or plasma vitamin E levels in untreated patients with thalassemia calculated for 11 to 12 termination in an average period was below the low normal levels while in treated patients the mean was 1.6+0.2 mg/100 ml (19). In the present study, vitamin E was ineffective in alteration of serum levels of Hb, Hct, RBC count which is consistent with previous reports.

Suthutvoravut U et al. pointed out that following supplementation, the plasma vitamin E increased but H_2_O_2_ hemolysis decreased to the normal values (20). In their study hematocrit did not change significantly which it is in agreement with our finding with our finding.

Theoretically, vitamin E and glutathione are the red cell protective antioxidants, and increasing levels of both should decrease intravascular hemolysis (21).In the present study, we found that plasma free hemoglobin, HCT and RBC count decreased after treating. This finding is, however, similar to what reported by Dissayabutra T et al. These investigators represented that this was due to plasma free iron, the main cause of oxidative stress, was not treated and there are other antioxidants involved in red blood cell protection (22).

Jamshidi et al studied in bilirubin after treating patients with vitamin E alone for 7 months and found no change. The authors concluded that total bilirubin (TB) after 3 months was insignificantly decreased (23).

Their results seems incompatible because our finding showed that after vitamin E treatment, the serum SGOT, SGPT and significantly SGOT were all reduced.

Bazvand F et al. showed that the bilirubin level in males was markedly higher than females in β-thalassemia patient (24).

Livrea M.A reported that no correlation existed between the age of the patients and hematological parameters, as well as any other parameter examined throughout that study which this is agree with our study (25). In a similar study , Dissayabutra et al represented that of both vitamin C and vitamin E had more benefit than supplementation of vitamin E alone (22). 

Prasad K et al reported that hypercholesterolemia decreased RBC count, Hct and Hb, but increased MCV, RDW, MCH, and MCHC, and had no effect on WBC and platelet counts, and MPV. Vitamin E did not affect any of the parameters of the hemopoietic system. Vitamin E does not affect the hemopoietic system during hypercholesterolemia (26).

Pfeifer W.P et al Investigated to study the effect of vitamin E treatment in oxidative stress of red and white blood cells in β-thalassaemia intermedia patients. In their study a significant reduction in reticulocyte count number was observed after vitamin E treatment. They reported that vitamin E treatment did not modify levels of hemoglobin. Although vitamin E is not capable of reducing anemia in these patients, it could be useful for reducing oxidative damage in other target organs in β-thalassaemic patients (27). An important point to note with our result was that Vitamin E had no effect on hemopoitic system parameters but effective on liver function test.

Attia M.M.A. studied the effects of antioxidant vitamins on antioxidant status and liver function in homozygous β-thalassemic patients. The patients were treated with vitamins E, for twelve months. After treatment, patients with β-thalassemia major exhibited significant improvements in the levels of non-enzymatic parameters as compared with the levels of these parameters before treatment. The ALT, AST levels in this study decreased significantly (28) and this result is compatible with our finding.
